# Physiology of lived experience: Cruising among the Swiss peaks

**DOI:** 10.1113/EP093451

**Published:** 2025-12-08

**Authors:** Grégoire P. Millet

**Affiliations:** ^1^ Institute of Sport Sciences, Faculty of Biology and Medicine University of Lausanne Lausanne Switzerland

On the starting line! The challenge ahead is intimidating: completing the SwissPeaks 700 (recently renamed the ‘Odyssey’), promoted as the ‘longest trail in the world’, at a distance of ∼700 km and with nearly 49 000 m of elevation gain. This mountain ultramarathon (MUM) forms a loop around the canton of Valais, Switzerland, beginning on the shores of Lake Geneva and extending to the Aletsch Glacier before returning to the start (Figures 1 and 2).

**FIGURE 1:**
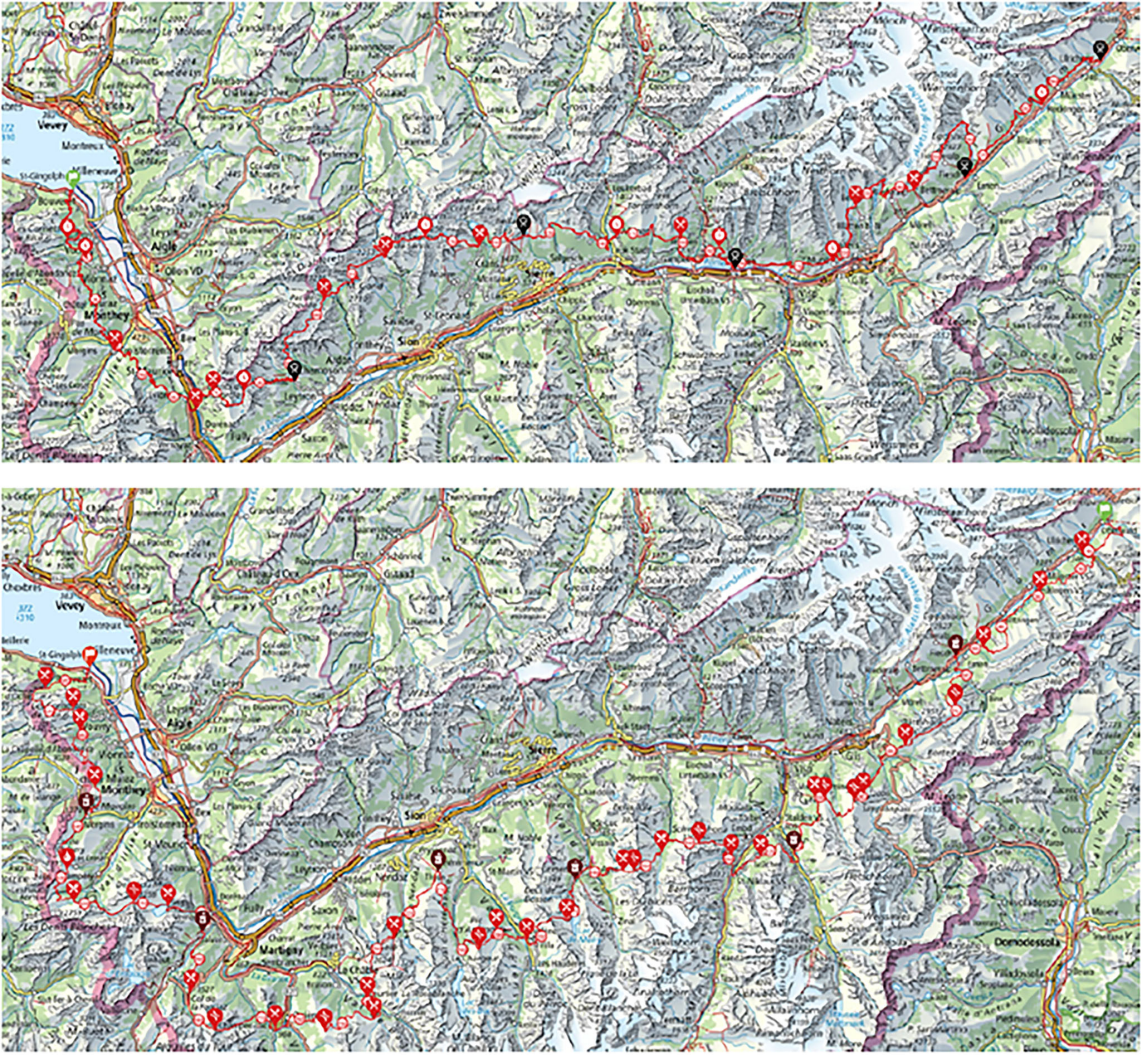
Race course of the SwissPeaks 700 mountain ultramarathon. The red symbols indicate the aid stations (food and drink). The black symbols show the location of the base camp (sleep possible).

**FIGURE 2:**
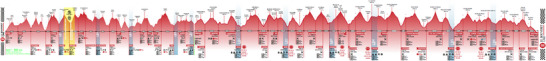
Race profile of the SwissPeaks 700 mountain ultramarathon.

I am the oldest participant on the start list (63 years old) among 100 ‘old horses’ selected for their extensive MUM experience (i.e., finishers of races >300 km with >20 000 m of elevation gain). I am well aware that practical experience and a bit of physiology knowledge derived from my work (as an exercise physiologist focusing on hypoxia and endurance) can offset the ‘biological clock’ and the progressive decline in maximal oxygen uptake with age only in part. This decay begins at ∼30–35 years of age and proceeds at ∼10% per decade (Astrand et al., 1973).

Nonetheless, I remain optimistic that my ‘live high, train low and high’ preparation (combining regular uphill/downhill walking in altitude and sleeping for 4 weeks in a hypoxic tent at a simulated altitude of ∼3000 m) would give me an edge. Beyond improving acclimatization (Millet & Jornet, 2019) and increasing haemoglobin mass (Kettunen et al., 2023), such preparation might also promote less well‐known peripheral adaptations linked to the compensatory vasodilatation induced by exercise in hypoxia (Casey & Joyner, 2012). Several factors highlight the importance of this last point:

1. Exercise intensity during such long MUMs is very low (typically 50%–60% of maximal oxygen uptake during ascents and probably even lower during descents; Savoldelli et al., 2017).
2. Running economy remains a key determinant of performance in MUMs, as in any endurance event (Millet, 2012), and depends strongly on neuromuscular factors (Barnes & Kilding, 2015).
3. A mismatch between oxygen delivery and consumption in working muscles (i.e., increased muscle deoxygenation) has been reported after MUMs (Vernillo et al., 2017).
4. Finally, in my case, age‐related increases in peripheral resistance further limit oxygen utilization (Capelli et al., 2026).

The specific demands of mountain ultramarathons also require careful preparation for the extensive downhill mileage and the associated eccentric loads that cause muscle damage and increase injury risk (Carmona et al., 2015), while negatively affecting microcirculation and leading to metabolic disturbances (Vernillo et al., 2017). However, such muscle‐damage concerns are somewhat mitigated in extremely long MUMs owing to the very low downhill velocities typically adopted. Indeed, relative muscle ‘preservation’ has been reported as race distance increases; for example, decrements in maximal voluntary contraction of the knee extensors and plantar flexors were smaller in a 330 km (D^+^: positive elevation gain 24 000 m) race than in a 170 km event (D^+^: positive elevation gain 10 000 m) (Saugy et al., 2013).

More concerning are the potential indirect health consequences of muscle damage (Scheer et al., 2022), including the risks of rhabdomyolysis (Rojas‐Valverde et al., 2020) and severe acute kidney injury (Belli et al., 2018; Irving et al., 1990), along with the possibility of persistent long‐term cardiac effects, such as myocardial fibrosis, arrhythmias or atrial fibrillation (Burtscher et al., 2022)

So, why do it? Why do we register and even pay for standing on the starting line, with no guarantee that we will be among the finishers a few days later? Is it the search for extreme physical and emotional experiences (Bill & Philippe, 2023) or the pursuit of personal goals and the satisfaction of achievement (Roebuck et al., 2018) or because this passion makes us happy when engaged in an open‐minded and non‐defensive manner (Bill et al., 2024)? Is it an innate psychological drive to explore our physical and mental limits (Roebuck et al., 2018)? External prestige? Or perhaps the fact that extremely long MUMs tend to be less competitive and more intrinsically goal oriented?

My own motivation is likely to draw from all these elements, combined with the desire to experience directly the physiological mechanisms that I study in my research.

Overall, the small ‘tribe’ gathered at the starting line appeared relaxed, although the magnitude of the challenge undoubtedly elicits fear and anxiety, particularly in the days preceding such an event (Urwin et al., 2021).

A final kiss, and we begin!

We begin with a long ascent (∼1000 m of positive elevation) that takes us from 375 to 1415 m. During this first climb, my priority is to pace myself wisely, resisting the collective momentum of the group by adopting the most appropriate velocity and step length. I am aware that my vertical velocity will inevitably decline climb after climb, day after day, with minimal change in step frequency but a progressive reduction in step length (Jeker et al., 2020).

I try to use my poles efficiently to reduce the mechanical load on my quadriceps and calf muscles, ‘saving my legs’ for what lies ahead (Giovanelli et al., 2023). This strategy promotes a longer, slower leg extension during push‐off and increases contact time, which is likely to reduce the rate of force production and provides metabolic benefits (Vernillo et al., 2014). When compared with uphill walking without poles, the combined effects, including reduced internal work, counterbalance the increased energy cost of involving both upper and lower limbs. Consequently, using poles at moderate intensity has minimal impact on overall metabolic expenditure while still reducing mechanical work (Giovanelli et al., 2019). This helps to explain why the vast majority of runners adopt poles in long mountain ultramarathons.

Night falls, but the first night outdoors does not yet bring significant sleep deprivation. Owing to workload and preparation for a scientific experiment, I had been unable to implement an effective ‘sleep banking’ strategy (i.e., increasing sleep duration in the days before the race) (Poussel et al., 2015). I am therefore slightly anxious about how sleep restriction might affect me during the forthcoming nights.

After several hours, in the middle of the night, only a scattered few runners remain visible across the mountains. I soon reach the first major challenge: a very steep descent of D^−^: negative gain 1300 m.

Like many ultra‐trail runners, who often present numerous low‐grade cartilage and meniscal lesions (Della Rosa et al., 2024), I had experienced chronic knee pain in the months leading up to the event (Diermeier et al., 2019; Knechtle & Nikolaidis, 2018). This had substantially reduced my running volume before the race. Having undergone meniscus surgery a few years earlier, I knew my knee would be a major limiting factor and my most likely reason for withdrawal. I therefore adopted an extremely conservative downhill pace, reducing joint load with careful pole use. Although limiting, this had been anticipated in my preparation, during which I deliberately targeted body‑weight reduction to decrease mechanical stress on the knee while also decreasing fat mass to improve my power‐to‐weight ratio, thereby enhancing uphill velocity (Lemire et al., 2021) and partly offsetting the inevitable reduction in downhill speed. This carefully controlled pacing strategy was essential, not only to remain comfortably ahead of intermediate cut‐off times but also to retain a buffer, should unforeseen problems slow me down later.

The first difficulties began after 2 days and ∼150 km of ascents and descents. I was feeling reasonably well, apart from some unpleasant nausea, which is a common exercise‐associated gastrointestinal symptom in ultra‐endurance events (Costa et al., 2025). We (Millet et al., 2025) recently hypothesized that the prevalence of exercise‐associated gastrointestinal symptoms is particularly high during the early phases of MUMs owing to a convergence of several factors, including excessive carbohydrate intake before or early in the race, pre‐race anxiety, inappropriate pacing, shifts between hypo‐ and hypervolaemia, altitude exposure and environmental temperature fluctuations. Of these six factors, none appeared especially relevant in my case. I consumed substantial protein rather than carbohydrates only; I felt confident with modest performance expectations; my pacing was deliberately protective; I was well acclimatized; and the weather (unfortunately) was consistently wet and cold. Although exercise‐associated gastrointestinal symptoms are a major cause of withdrawal from ultramarathons (Costa et al., 2016), the origin of my nausea over these days, and why it eventually resolved, remain unclear. Certainly, an area for future investigation!

Between 350 and 380 km, despite several relatively long sleep periods (averaging 5 h per night), my physical energy (and parallel motivation) declined markedly, owing to persistent knee pain and relentless poor weather (fog, cold and rain).

Ultrarunners are known to have a high tolerance to pain, partly attributable to reduced pain‑related anxiety and fewer avoidance behaviours; pain is ‘part of the game’ rather than a threat. Mental toughness scores are higher among ultramarathon runners than in other sports (Brace et al., 2020). I might be an exception regarding this particular psychological faculty.

My options were limited: stop and sleep (never withdraw without first attempting a nap, because you might change your mind afterwards); withdraw; or engage in a targeted pain‐specific resilience strategy. Fortunately, Laurent (a highly experienced friend) ‘dragged’ me slowly all the way to Lengritz (2416 m), where hot drinks restored my morale. Beyond his words, ‘We move slow but we keep moving’, this anecdote confirms the importance of the social interactions (in this case, with fellow runners) on the response of the athlete to MUM‐related stressors (Harman et al., 2019). Functional ‘teams’ arise spontaneously, offering reciprocal support. In mountain sports, such social relationships are mainly instrumentally oriented and valuable for coping with both physical and environmental challenges. Such complementary alliances proved equally important at ∼580 km, at the technical pass of ‘fenêtre d'Arpette’.

From pass to pass and valley to valley, the cumulative distance increases, with the most demanding sections still ahead. Favourable weather is forecast, and the anticipated psychological benefit of reuniting with my daughters on the trail approaches (Figure 3). Having contact with family members helps to cope with anxiety when the runner experiences difficulties (Harman et al., 2019). The psychological and emotional support that my family provided, particularly given that they had travelled a long distance to be present, was exceptional. Such affective social interactions play a key role in helping athletes to cope with the demands of an activity often perceived as solitary (Harman et al., 2019).

**FIGURE 3:**
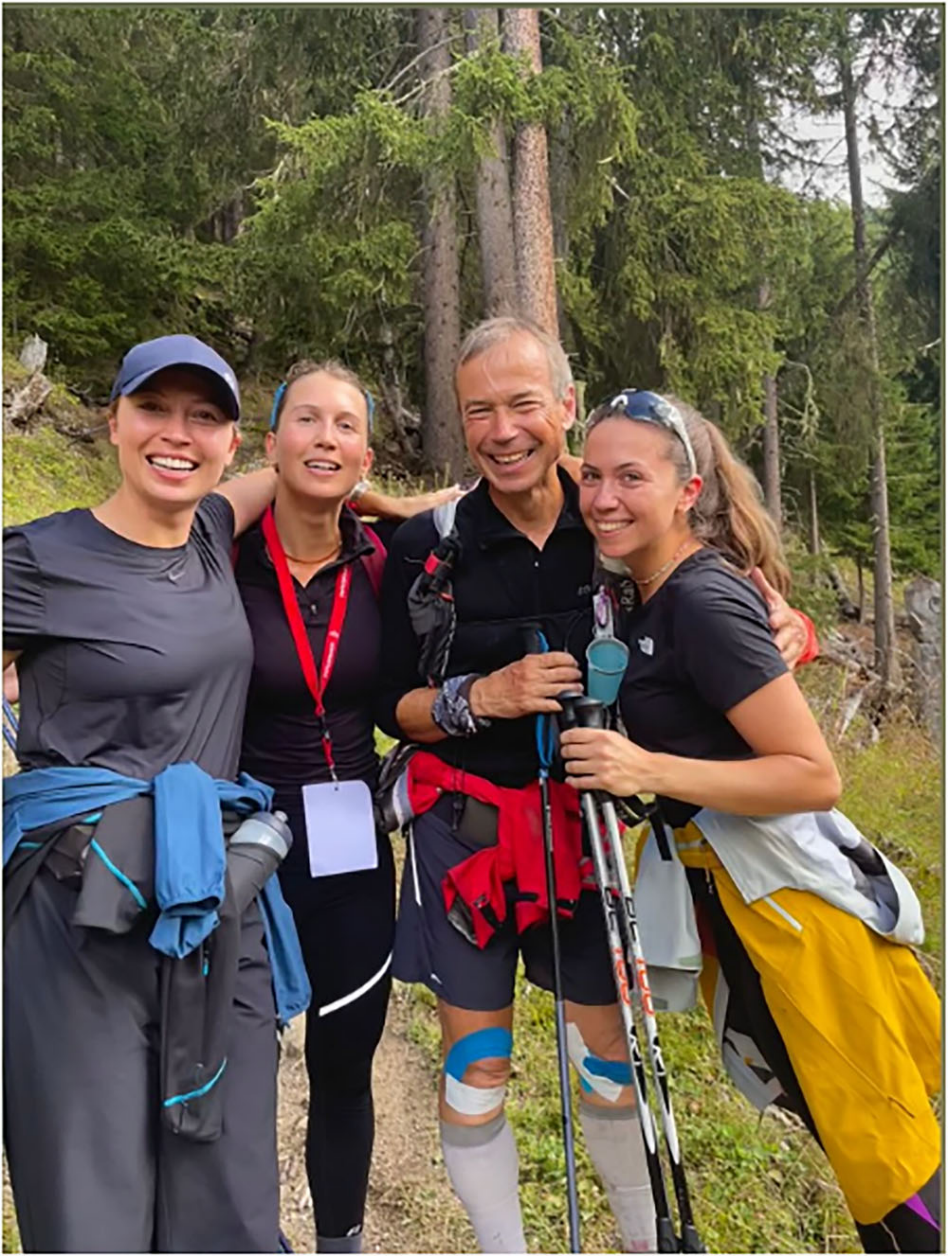
Support from loved ones is crucial for coping with the challenges of the race. Notice the oedema on the runner's face.

Around 500 km, I began to experience substantial fluid retention, with progressively increasing oedemas visible in the lower limbs and facial region (Figure 3). This resulted in a substantial increase in body mass between the start and the finish of the race. Oedemas are commonly reported during ultramarathons (Bracher et al., 2012; Vitiello et al., 2015; Zanchi et al., 2016) and are the observable consequences of the increase in total body water (Knechtle et al., 2008), including plasma volume (i.e., pronounced hypervolaemia) (Krumm et al., 2025). I had not experienced this phenomenon during my previous MUMs, and the underlying physiological mechanisms remain unclear. Given that pro‐inflammatory responses can contribute to hypervolaemic processes (Robach et al., 2014), it cannot be ruled out that the chronic knee pain experienced during the race acted as a primary trigger. The consequences for renal function might be substantial and remain understood only in part. In contrast, the short‐term effects (Linder et al., 2025; Pasternak et al., 2023; Vauthier et al., 2024) and, potentially, long‐term effects (Hodgson et al., 2017; Rojas‐Valverde et al., 2020) of hypohydration‐induced acute kidney injury are better documented, including its possible association with cardiac dysfunction (Burtscher et al., 2022).

The emotional impact at the finish line was profound, and the journey across the Swiss peaks proved highly instructive. As a physiologist, directly experiencing both the advantages and the drawbacks of such an extreme MUM provides valuable insights that might help to refine my understanding of the associated physiological stressors. This experiential perspective could contribute to the development of strategies aimed at mitigating the deleterious consequences of prolonged MUMs (e.g., sleep deprivation, inflammatory responses, pronounced hypervolaemia and oedemas, and low‐grade knee cartilage damage), while simultaneously acknowledging and optimizing the beneficial adaptations elicited before and during the race, mainly the increased level of physical activity (Stamatakis et al., 2025; Tarp et al., 2024).

To conclude, in my view, few stimuli offer a more powerful health‐promoting leverage than sustained moderate‐intensity exercise in mountainous environments, because it combines the well‐established benefits of green exercise (Laezza et al., 2025; Pretty et al., 2005) and those of moderate‐altitude exposure (Burtscher et al., 2024; Mallet et al., 2026).

How green it was, how slow it was, and what a long period of exercise at altitude it turned out to be! (Figure 4). We were truly fortunate to have such an adventure right on our doorstep.

**FIGURE 4:**
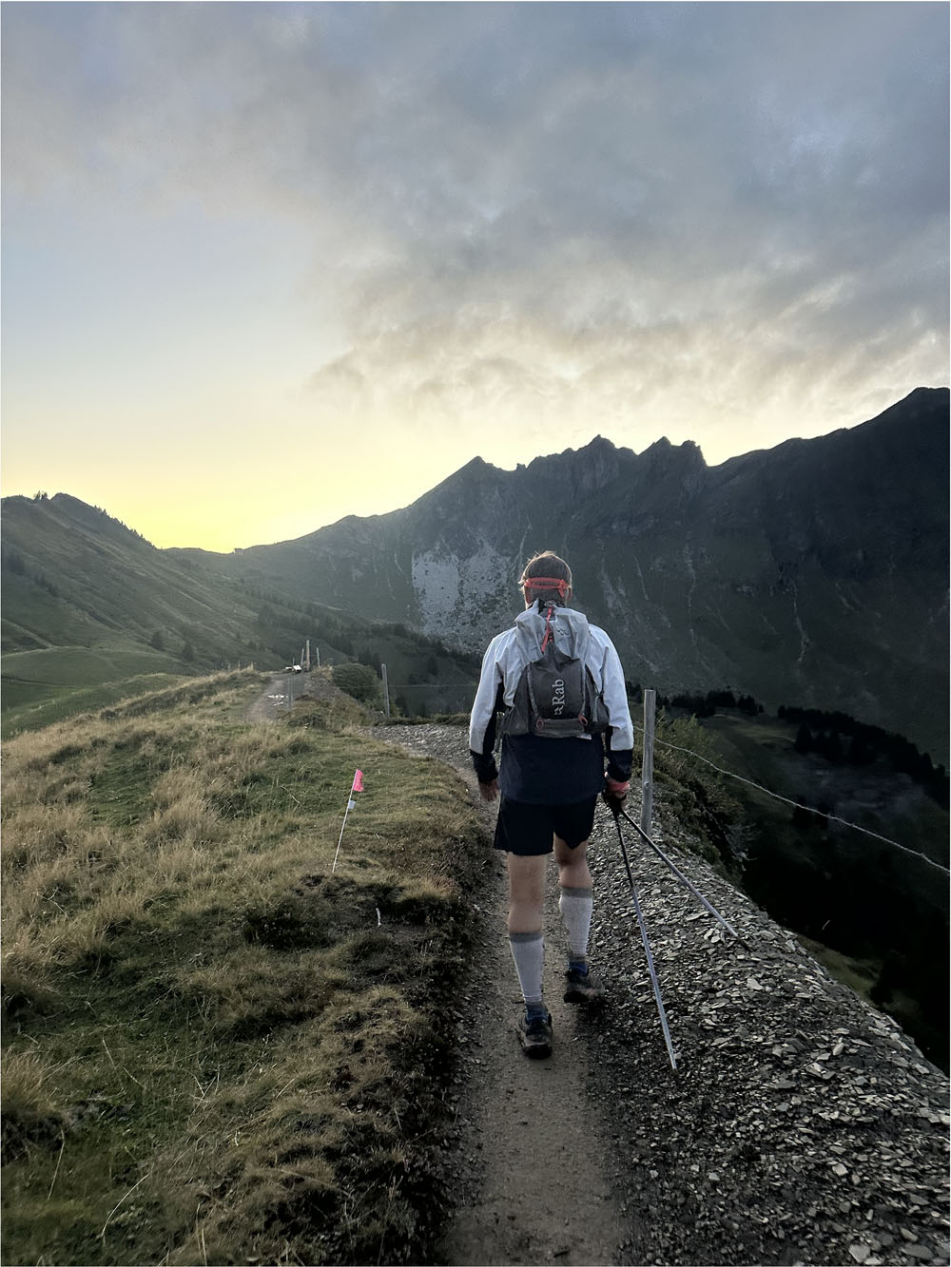
Green exercise in a mountainous environment.

## AUTHOR CONTRIBUTIONS

Sole author.

## CONFLICT OF INTEREST

None declared.

## FUNDING INFORMATION

None.

## References

[eph70162-bib-0001] Astrand, I. , Astrand, P. O. , Hallback, I. , & Kilbom, A. (1973). Reduction in maximal oxygen uptake with age. Journal of Applied Physiology, 35, 649–654. 4770349 10.1152/jappl.1973.35.5.649

[eph70162-bib-0002] Barnes, K. R. , & Kilding, A. E. (2015). Strategies to improve running economy. Sports Medicine, 45, 37–56. 25164465 10.1007/s40279-014-0246-y

[eph70162-bib-0003] Belli, T. , Macedo, D. V. , de Araujo, G. G. , Dos Reis, I. G. M. , Scariot, P. P. M. , Lazarim, F. L. , Nunes, L. A. S. , Brenzikofer, R. , & Gobatto, C. A. (2018). Mountain ultramarathon induces early increases of muscle damage, inflammation, and risk for acute renal injury. Frontiers in Physiology, 9, 1368. 30349484 10.3389/fphys.2018.01368 PMC6186806

[eph70162-bib-0004] Bill, T. , Dessart, G. , & Antonini Philippe, R. (2024). Does ultra‐endurance passion make athletes happy? Sports, 12, 149. 38921843 10.3390/sports12060149 PMC11209472

[eph70162-bib-0005] Bill, T. , & Philippe, R. A. (2023). A new temporal framework for the passionate engagement journey of ultra‐endurance athletes: A qualitative investigation. PLoS ONE, 18, e0293864. 38011082 10.1371/journal.pone.0293864 PMC10681185

[eph70162-bib-0006] Brace, A. W. , George, K. & , & Lovell, G. P. (2020). Mental toughness and self‐efficacy of elite ultra‐marathon runners. PLoS ONE, 15, e0241284. 33147236 10.1371/journal.pone.0241284 PMC7641431

[eph70162-bib-0007] Bracher, A. , Knechtle, B. , Gnadinger, M. , Burge, J. , Rust, C. A. , Knechtle, P. , & Rosemann, T. (2012). Fluid intake and changes in limb volumes in male ultra‐marathoners: Does fluid overload lead to peripheral oedema? European Journal of Applied Physiology & Occupational Physiology, 112, 991–1003. 21720884 10.1007/s00421-011-2056-3

[eph70162-bib-0008] Burtscher, J. , Citherlet, T. , Camacho‐Cardenosa, A. , Camacho‐Cardenosa, M. , Raberin, A. , Krumm, B. , Hohenauer, E. , Egg, M. , Lichtblau, M. , Muller, J. , Rybnikova, E. A. , Gatterer, H. , Debevec, T. , Baillieul, S. , Manferdelli, G. , Behrendt, T. , Schega, L. , Ehrenreich, H. , Millet, G. P. , … Mallet, R. T. (2024). Mechanisms underlying the health benefits of intermittent hypoxia conditioning. The Journal of Physiology, 602, 5757–5783. 37860950 10.1113/JP285230

[eph70162-bib-0009] Burtscher, J. , Vanderriele, P. E. , Legrand, M. , Predel, H. G. , Niebauer, J. , O'Keefe, J. H. , Millet, G. P. , & Burtscher, M. (2022). Could repeated cardio‐renal injury trigger late cardiovascular sequelae in extreme endurance athletes? Sports Medicine, 52, 2821–2836. 35851948 10.1007/s40279-022-01734-8 PMC9691495

[eph70162-bib-0010] Capelli, C. , Ferretti, G. , di Prampero, P. E. , & Tam, E. (2026). Cardiovascular and peripheral factors affecting the decay of maximal oxygen uptake across the spectrum of age in humans. European Journal of Applied Physiology, 126, 1635–1643. 41160092 10.1007/s00421-025-06031-6

[eph70162-bib-0011] Carmona, G. , Roca, E. , Guerrero, M. , Cusso, R. , Irurtia, A. , Nescolarde, L. , Brotons, D. , Bedini, J. L. , & Cadefau, J. A. (2015). Sarcomere disruptions of slow fiber resulting from mountain ultramarathon. International Journal of Sports Physiology and Performance, 10, 1041–1047. 25848839 10.1123/ijspp.2014-0267

[eph70162-bib-0012] Casey, D. P. , & Joyner, M. J. (2012). Compensatory vasodilatation during hypoxic exercise: Mechanisms responsible for matching oxygen supply to demand. The Journal of Physiology, 590, 6321–6326. 22988134 10.1113/jphysiol.2012.242396 PMC3533194

[eph70162-bib-0013] Costa, R. J. , Snipe, R. , Camoes‐Costa, V. , Scheer, V. , & Murray, A. (2016). The impact of gastrointestinal symptoms and dermatological injuries on nutritional intake and hydration status during ultramarathon events. Sports Medicine Open, 2, 16. 26767151 10.1186/s40798-015-0041-9 PMC4701764

[eph70162-bib-0014] Costa, R. J. S. , Gaskell, S. K. , Henningsen, K. , Jeacocke, N. A. , Martinez, I. G. , Mika, A. , Scheer, V. , Scrivin, R. , Snipe, R. M. J. , Wallett, A. M. , & Young, P. (2025). Sports dietitians Australia and ultra sports science foundation joint position statement: a practitioner guide to the prevention and management of exercise‐associated gastrointestinal perturbations and symptoms. Sports Medicine, 55(5), 1097–1134. 40195264 10.1007/s40279-025-02186-6 PMC12106582

[eph70162-bib-0015] Della Rosa, T. , Gaulin, B. , Schwach, M. , Gaillot, J. , Pailhe, R. , & Horteur, C. (2024). Evaluation of the impact of ultra‐trail running on knee cartilage using magnetic resonance imaging t2 mapping. Journal of Sports Medicine & Physical Fitness, 64, 1321–1328. 39268771 10.23736/S0022-4707.24.15966-X

[eph70162-bib-0016] Diermeier, T. , Beitzel, K. , Bachmann, L. , Petersen, W. , Esefeld, K. , Wortler, K. , Imhoff, A. B. , & Achtnich, A. (2019). Mountain ultramarathon results in temporary meniscus extrusion in healthy athletes. Knee Surgery, Sports Traumatology, Arthroscopy, 27, 2691–2697. 10.1007/s00167-018-5303-x 30465096

[eph70162-bib-0017] Giovanelli, N. , Pellegrini, B. , Bortolan, L. , Mari, L. , Schena, F. , & Lazzer, S. (2023). Do poles really “save the legs” during uphill pole walking at different intensities? European Journal of Applied Physiology, 123, 2803–2812. 37392255 10.1007/s00421-023-05254-9

[eph70162-bib-0018] Giovanelli, N. , Sulli, M. , Kram, R. , & Lazzer, S. (2019). Do poles save energy during steep uphill walking? European Journal of Applied Physiology, 119, 1557–1563. 31020400 10.1007/s00421-019-04145-2

[eph70162-bib-0019] Harman, B. , Kosirnik, C. , & Antonini Philippe, R. (2019). From social interactions to interpersonal relationships: Influences on ultra‐runners' race experience. PLoS ONE, 14, e0225195. 31790446 10.1371/journal.pone.0225195 PMC6886831

[eph70162-bib-0020] Hodgson, L. E. , Walter, E. , Venn, R. M. , Galloway, R. , Pitsiladis, Y. , Sardat, F. , & Forni, L. G. (2017). Acute kidney injury associated with endurance events‐is it a cause for concern? A systematic review. British Medical Journal Open Sport Exercise Medicine, 3, e000093. 10.1136/bmjsem-2015-000093 PMC5731225 29259804

[eph70162-bib-0021] Irving, R. A. , Noakes, T. D. , Burger, S. C. , Myburgh, K. H. , Querido, D. , & van, Z. , & Smit, R. (1990). Plasma volume and renal function during and after ultramarathon running. Medicine & Science in Sports & Exercise, 22, 581–587. 2233195 10.1249/00005768-199010000-00007

[eph70162-bib-0022] Jeker, D. , Falbriard, M. , Vernillo, G. , Meyer, F. , Savoldelli, A. , Degache, F. , Schena, F. , Aminian, K. , & Millet, G. P. (2020). Changes in spatio‐temporal gait parameters and vertical speed during an extreme mountain ultra‐marathon. European Journal of Sport Science, 20, 1339–1345. 31914356 10.1080/17461391.2020.1712480

[eph70162-bib-0023] Kettunen, O. , Leppavuori, A. , Mikkonen, R. , Peltonen, J. E. , Nummela, A. , Wikstrom, B. , & Linnamo, V. (2023). Hemoglobin mass and performance responses during 4 weeks of normobaric “live high‐train low and high”. Scandinavian Journal of Medicine & Science in Sports, 33, 1335–1344. 37114394 10.1111/sms.14378

[eph70162-bib-0024] Knechtle, B. , Duff, B. , Schulze, I. , & Kohler, G. (2008). A Multi‐Stage Ultra‐Endurance Run over 1,200 KM Leads to a Continuous Accumulation of Total Body Water. Journal of Sports Science and Medicine, 7, 357–364. 24149903 PMC3761892

[eph70162-bib-0025] Knechtle, B. , & Nikolaidis, P. T. (2018). Physiology and pathophysiology in ultra‐marathon running. Frontiers in Physiology, 9, 634. 29910741 10.3389/fphys.2018.00634 PMC5992463

[eph70162-bib-0026] Krumm, B. , Raberin, A. , Citherlet, T. , Tagliapietra, G. , Faiss, R. , Pialoux, V. , Debevec, T. , Giardini, G. , & Millet, G. P. (2025). Accelerated red blood cell turnover following extreme mountain ultramarathon? Medicine & Science in Sports & Exercise, 57, 904–911. 39629720 10.1249/MSS.0000000000003621 PMC12147757

[eph70162-bib-0027] Laezza, L. , Vacondio, M. , Fornasiero, A. , Pellegrini, B. , Pasini, M. , Brondino, M. , & De Dominicis, S. (2025). Evaluating the benefits of green exercise: A randomized controlled trial in natural and built environments assessed for their restorative properties. Psychology Sport and Exercise, 80, 102883. 10.1016/j.psychsport.2025.102883 40403944

[eph70162-bib-0028] Lemire, M. , Hureau, T. J. , Favret, F. , Geny, B. , Kouassi, B. Y. L. , Boukhari, M. , Lonsdorfer, E. , Remetter, R. , & Dufour, S. P. (2021). Physiological factors determining downhill vs uphill running endurance performance. Journal of Science and Medicine in Sport, 24, 85–91. 32646746 10.1016/j.jsams.2020.06.004

[eph70162-bib-0029] Linder, B. A. , Jeong, S. , Stute, N. L. , El‐Kurd, O. B. , Vondrasek, J. D. , Pasternak, A. , Bagley, J. R. , Watso, J. C. , Schlader, Z. J. , Babcock, M. C. , Grosicki, G. J. , & Robinson, A. T. (2025). Hypohydration augments the acute increase in urinary biomarkers of kidney injury following the 100‐mile western states endurance run. Journal of Applied Physiology, 139(5), 1379–1391. 41115071 10.1152/japplphysiol.00289.2025 PMC12765470

[eph70162-bib-0030] Mallet, R. T. , Burtscher, J. , Gatterer, H. , Hufner, K. , Verges, S. , Debevec, T. , & Burtscher, M. (2026). Reduced cardiovascular mortality at moderate altitude: A putative role of physical activity and body mass. The Journal of Physiology . Advance online publication. 10.1113/JP290022 41178785

[eph70162-bib-0031] Millet, G. P. (2012). Economy is not sacrificed in ultramarathon runners. Journal of Applied Physiology, 113, 686. 22896682 10.1152/japplphysiol.00642.2012

[eph70162-bib-0032] Millet, G. P. , & Jornet, K. (2019). On top to the top‐acclimatization strategy for the “fastest known time” to Mount Everest. International Journal of Sports Physiology and Performance, 14, 1438–1441. 30958056 10.1123/ijspp.2018-0931

[eph70162-bib-0033] Millet, G. P. , Raberin, A. , & Krumm, B. (2025). Comment on: “Sports dietitians Australia and Ultra Sports Science Foundation joint position statement”: A practitioner guide to the prevention and management of exercise‐associated gastrointestinal perturbations and symptoms. Sports Medicine . 55, (11), 2933–2935. 10.1007/s40279-025-02274-7 40730939

[eph70162-bib-0034] Pasternak, A. V. , Newkirk‐Thompson, C. , Howard, J. H., 3rd , Onate, J. C. , & Hew‐Butler, T. (2023). Four Cases of Acute Kidney Injury Requiring Dialysis in Ultramarathoners. Wilderness & Environmental Medicine, 34, 218–221. 36805094 10.1016/j.wem.2022.12.004

[eph70162-bib-0035] Poussel, M. , Laroppe, J. , Hurdiel, R. , Girard, J. , Poletti, L. , Thil, C. , Didelot, A. , & Chenuel, B. (2015). Sleep Management Strategy and Performance in an Extreme Mountain Ultra‐marathon. Research in Sports Medicine, 23, 330–336. 26020095 10.1080/15438627.2015.1040916

[eph70162-bib-0036] Pretty, J. , Peacock, J. , Sellens, M. , & Griffin, M. (2005). The mental and physical health outcomes of green exercise. International Journal of Environmental Health Research, 15, 319–337. 16416750 10.1080/09603120500155963

[eph70162-bib-0037] Robach, P. , Boisson, R. C. , Vincent, L. , Lundby, C. , Moutereau, S. , Gergele, L. , Michel, N. , Duthil, E. , Feasson, L. , & Millet, G. Y. (2014). Hemolysis induced by an extreme mountain ultra‐marathon is not associated with a decrease in total red blood cell volume. Scandinavian Journal of Medicine & Science in Sports, 24, 18–27. 22672635 10.1111/j.1600-0838.2012.01481.x

[eph70162-bib-0038] Roebuck, G. S. , Fitzgerald, P. B. , Urquhart, D. M. , Ng, S.‐K. , Cicuttini, F. M. , & Fitzgibbon, B. M. (2018). The psychology of ultra‐marathon runners: A systematic review. Psychology of Sport and Exercise, 37, 43–58.

[eph70162-bib-0039] Rojas‐Valverde, D. , Sanchez‐Urena, B. , Crowe, J. , Timon, R. , & Olcina, G. J. (2020). Exertional rhabdomyolysis and acute kidney injury in endurance sports: A systematic review. European Journal of Sport Science, 21(2), 261–274. 32202487 10.1080/17461391.2020.1746837

[eph70162-bib-0040] Saugy, J. , Place, N. , Millet, G. Y. , Degache, F. , Schena, F. , & Millet, G. P. (2013). Alterations of neuromuscular function after the world's most challenging mountain ultra‐marathon. PLoS ONE, 8, e65596. 23840345 10.1371/journal.pone.0065596 PMC3694082

[eph70162-bib-0041] Savoldelli, A. , Fornasiero, A. , Trabucchi, P. , Limonta, E. , La Torre, A. , Degache, F. , Pellegrini, B. , Millet, G. P. , Vernillo, G. , & Schena, F. (2017). the energetics during the world's most challenging mountain ultra‐marathon‐a case study at the Tor des Geants(R). Frontiers in Physiology, 8, 1003. 29259560 10.3389/fphys.2017.01003 PMC5723401

[eph70162-bib-0042] Scheer, V. , Tiller, N. B. , Doutreleau, S. , Khodaee, M. , Knechtle, B. , Pasternak, A. , & Rojas‐Valverde, D. (2022). Potential long‐term health problems associated with ultra‐endurance running: A narrative review. Sports Medicine, 52, 725–740. 34542868 10.1007/s40279-021-01561-3 PMC8450723

[eph70162-bib-0043] Stamatakis, E. , Biswas, R. K. , Koemel, N. A. , Sabag, A. , Pulsford, R. , Atkin, A. J. , Stathi, A. , Cheng, S. , Thogersen‐Ntoumani, C. , Blodgett, J. M. , Bauman, A. , Celis‐Morales, C. , Hamer, M. , Gill, J. M. R. , & Ahmadi, M. N. (2025). Dose response of incidental physical activity against cardiovascular events and mortality. Circulation, 151, 1063–1075. 40228066 10.1161/CIRCULATIONAHA.124.072253 PMC12002041

[eph70162-bib-0044] Tarp, J. , Dalene, K. E. , Fagerland, M. W. , Steene‐Johannesen, J. , Hansen, B. H. , Anderssen, S. A. , Hagstromer, M. , Dohrn, I. M. , Dempsey, P. C. , Wijndaele, K. , Brage, S. , Nordstrom, A. , Nordstrom, P. , Diaz, K. M. , Howard, V. J. , Hooker, S. P. , Morseth, B. , Hopstock, L. A. , Sagelv, E. H. , … Ekelund, U. (2024). Physical activity volume, intensity, and mortality: Harmonized meta‐analysis of prospective cohort studies. American Journal of Preventive Medicine, 67, 887–896. 39089430 10.1016/j.amepre.2024.07.022

[eph70162-bib-0045] Urwin, C. S. , Main, L. C. , Mikocka‐Walus, A. , Skvarc, D. R. , Roberts, S. S. H. , Condo, D. , Carr, A. J. , Convit, L. , Jardine, W. , Rahman, S. S. , & Snipe, R. M. J. (2021). The relationship between psychological stress and anxiety with gastrointestinal symptoms before and during a 56 km ultramarathon running race. Sports Medicine Open, 7, 93. 34897557 10.1186/s40798-021-00389-5 PMC8665950

[eph70162-bib-0046] Vauthier, J. C. , Touze, C. , Mauvieux, B. , Hingrand, C. , Delaunay, P. L. , Besnard, S. , Jouffroy, R. , Noirez, P. , Maboudou, P. , Parent, C. , Heyman, E. , & Poussel, M. (2024). Increased risk of acute kidney injury in the first part of an ultra‐trail‐Implications for abandonment. Physiological Reports, 12, e15935. 38684379 10.14814/phy2.15935 PMC11058001

[eph70162-bib-0047] Vernillo, G. , Brighenti, A. , Limonta, E. , Trabucchi, P. , Malatesta, D. , Millet, G. P. , & Schena, F. (2017). Effects of ultratrail running on skeletal‐muscle oxygenation dynamics. International Journal of Sports Physiology and Performance, 12, 496–504. 27617750 10.1123/ijspp.2015-0745

[eph70162-bib-0048] Vernillo, G. , Savoldelli, A. , Zignoli, A. , Trabucchi, P. , Pellegrini, B. , Millet, G. P. , & Schena, F. (2014). Influence of the world's most challenging mountain ultra‐marathon on energy cost and running mechanics. European Journal of Applied Physiology, 114, 929–939. 24477570 10.1007/s00421-014-2824-y

[eph70162-bib-0049] Vitiello, D. , Degache, F. , Saugy, J. J. , Place, N. , Schena, F. , & Millet, G. P. (2015). The increase in hydric volume is associated to contractile impairment in the calf after the world's most extreme mountain ultra‐marathon. Extreme Physiology & Medicine, 4, 18. 26500765 10.1186/s13728-015-0037-6 PMC4618124

[eph70162-bib-0050] Zanchi, D. , Viallon, M. , Le Goff, C. , Millet, G. P. , Giardini, G. , Croisille, P. , & Haller, S. (2016). Extreme mountain ultra‐marathon leads to acute but transient increase in cerebral water diffusivity and plasma biomarkers levels changes. Frontiers in Physiology, 7, 664. 28105018 10.3389/fphys.2016.00664 PMC5214892

